# The role of farmers’ green values in creation of green innovative intention and green technology adoption behavior: Evidence from farmers grain green production

**DOI:** 10.3389/fpsyg.2022.980570

**Published:** 2022-10-14

**Authors:** Risheng Gao, Haitao Zhang, Chunming Gong, Zhihua Wu

**Affiliations:** ^1^Jiangxi Regional Development Research Institute, Jiangxi University of Technology, Nanchang, China; ^2^School of Economics and Management, Jiangxi Agricultural University, Nanchang, China

**Keywords:** farmer green values, green innovative intention, green technology adoption behavior, health consciousness, organizational behavior

## Abstract

Grain for Green Project (GGP) is one of China’s important ecological restoration projects. The key rationale of this Program is to decrease soil erosion and develop ecological conditions. The agricultural sector is putting efforts to promote green innovation and production among farmers to achieve the targets of ecological restoration projects. However, farmers’ green values could play a constructive role in building green innovative intention and green technology adoption behaviors. Based on the unified theory of acceptance and use of technology (UTAUT), the present study investigates the association between farmers’ green values and green technology adoption behavior. For empirical investigation, the current study assumes that farmers’ green values positively correlate with green innovative intention and green technology adoption behavior, respectively. Further, this study proposes that green innovative intention positively impacts green technology adoption behavior. The present study also aims to determine the mediating role of green innovative intention in the relationship between farmer green values and green technology adoption behavior. This study also attempts to check the moderating role of health consciousness in the relationship between farmer green values and green innovative intention and the relationship between farmer green values and green technology adoption behavior, respectively. For empirical analyses, the present study gathered data from303 farmers in China through a structured questionnaire method using a convenient sampling technique. The present study applied partial least square structural equation modeling for empirically examining hypotheses using Smart PLS software. The findings confirmed that farmers’ green values have a positive association with green innovative intention and green technology adoption behavior, respectively. The results further verified that green innovative intention positively correlates with green technology adoption behavior. The finding also authenticated that green innovative intention positively mediates the relationship between farmers’ green values and green technology adoption behavior. The moderating role of health consciousness in the relationship between farmers’ green values and green innovative intention is also confirmed by the results of this study. However, the findings revealed that health consciousness does not moderate the relationship between farmers’ green values and green technology adoption behavior. This study’s findings serve the literature by providing empirical insight on the importance of farmers’ green values for green innovative intention and green technology adoption behavior. Moreover, the findings also have important theoretical and practical implications.

## Introduction

Agriculture has a pivotal position in the conversation about reducing the effects of climate change and finding ways to adapt to those changes for several reasons (Porter et al., 2014). First, a significant proportion of the world’s population continues to make their living through agricultural activities. Agriculture is one of the economic sectors contributing to a detrimental impact on human health and the environment (Rockström et al., 2017). Secondly, agriculture is one of the sectors of the economy that is most susceptible to the effects of climate change. For example, agriculture is one of the most significant sectors responsible for releasing greenhouse gases (GHG), particularly in developing nations, where this industry is responsible for an average of 35% of all GHG emissions (Rockström et al., 2017).

Most people agree that safeguarding the environment and enhancing food safety are vital goals closely related to small farmers’ daily lives. Governments and public organizations are making significant efforts to limit the use of chemicals in agricultural operations (Kumar et al., 2019). These compounds have been found in various foods, including vegetables, fruits, and beverages (Fan et al., 2014). As a result, having strong food safety regulations has become crucial for ensuring food security. China was the world’s biggest user of pesticides, according to Food and Agriculture Organization (FAO; Fan et al., 2014). About 0.067 kg/ha of pesticides are used annually, a higher consumption rate than in certain wealthy nations. However, only 35% of pesticides are used effectively, well below the 50–60% norm seen in wealthy nations (Grung et al., 2015).

It is alarming as the use of chemicals impacts the health of farmers and end users. There is now a renewed emphasis on developing green innovations due to recent worries about climate change, carbon emissions, degradation of natural capital, and the broader public interest in green consumption (Liu et al., 2017; Lhotka et al., 2018). Green innovations are the new trend in fighting the use of chemicals and pesticides in crops. Therefore, policymakers and the corporate sector see building (green) innovation capacity as crucial to a shift to sustainable development (Schaltegger et al., 2017). The term green innovation refers to any process or product that is innovative both in and of itself. Once it is integrated into an existing system, the environmental sustainability of manufacturing activity may be improved (Schiederig et al., 2012).

All cutting-edge innovations that may be integrated into the production process, such as agricultural machinery and irrigation systems, are categorized as green technological innovations. Green innovations, such as recycling agricultural waste (He et al., 2016), relating to the employment of novel concepts, procedures, and methods in farming. In conclusion, green organizational innovations refer either to alterations in the organizational structure of a farm business or to various organizational schemes among various players specializing in the manufacturing and supply of agricultural goods. These alterations might occur inside a farm enterprise or during the production and provision of agricultural products (Tyfield et al., 2015).

Green innovations in agriculture may be categorized as technical, administrative, and organizational. Technological green innovations help in reducing workload of farmers without harming the environment. These technological green innovations include use of geographic innovation systems (GIS). This is based on computer operated process of analysis on geographically referenced information about crops and farmlands. Similarly, global positioning system (GPS) is also a technological tool for green innovations as it helps in easing down the farmers’ operations even in zero visibility (Ramaano, 2022). The administrative type of green innovations in agriculture deals with administered usage of chemical fertilizers and pesticides.

While organizational type of green innovations is more concerned with setup of institutes and repositories for keeping the farmland records along-with providing farm-based environmental goods to the farming community (Dudek and Wrzaszcz, 2020). When used in the production process, all three of these forms of innovation may reduce the environmental impact of agricultural practices while also boosting or sustaining farm earnings and contributing to the well-being of farmers (He et al., 2016). There is a need to inculcate the green values in farmers’ perceptions of agriculture. It would add to the green innovation intentions of farmers. There is a need to incorporate these green innovations into farmers’ intentions. Once farmers have developed the intentions of using green innovations, it may help adopt green technology (Krishnan et al., 2021). All these efforts will ultimately curb the spread of chemical usage in the agriculture sector of China leading to sustainable systems of production.

There is an assumption that incorporating environmentally friendly technologies into farming lessens how much damage agriculture does to the environment and raises the sustainability of agricultural systems. According to a study conducted in Italy, the use of bobbing hydroponic systems can reduce the environmental impact of urban farming, whereas other technological advancements like solar air heaters and photo-voltaic pumping systems also lessen the adverse environmental effects of greenhouse farming (Sanyé-Mengual et al., 2015; Hassanien et al., 2016). However, technology adoption also maintains the agricultural industry’s financial stability, which is crucial for the survival of small-scale farmers. In support of this claim, Pretty et al. (2011) discovered that the adoption of environmentally friendly technologies such as integrated pest control or conservation measures resulted in an increase of more than twice in the agricultural yields of African farmers.

In agricultural innovation research, the question of the intentions behind farmers’ adoption or rejection of technologies remains crucial and significant. Green and innovative technologies are anticipated to substantially impact food security, agricultural sector climate change mitigation, and adaptation (Rockström et al., 2017). When it comes to the issue of climate change, in particular, some of the most promising technologies may be characterized as green innovative technologies. These technologies consist of procedures or materials that lessen the adverse effects on the environment. However, using modern technology is a prerequisite for taking advantage of their promises. Adopting technological innovations is the basis for their final worth (Bukchin and Kerret, 2018).

It might be challenging to pinpoint tactics for enhancing innovation policy without understanding what motivates the adoption of green technologies. However, since the emphasis is often on specific intents that facilitate the dissemination process, this area’s work has remained too far (Lioutas and Charatsari, 2018). For instance, research suggests that farmers’ decisions to accept innovations are driven by economic considerations or, in the case of green innovations, by their social orientation and concern. However, the location of farmers in rural networks and the amount of knowledge they possess also have a significant impact (Lioutas and Charatsari, 2018).

The farmers’ concerns and behaviors about green innovations are referred to as green values. Green values are quite important as these may not only develop green intentions among farmers but these values are also crucial for molding behaviors of farmers toward adoption of green technologies. Moreover, green intentions would also direct the green technology adoption behaviors in farmers. These farmer behaviors are grounded based on the Theory of Planned Behaviors (TPB), Technology Acceptance Model (TAM), and Theory of Acceptance and Use of Technology (TAUT; Bukchin and Kerret, 2018). By adopting green technology, farmers’ behaviors are modified by developing green values which develop their innovative intentions. In this way, farmers will develop technology adoption behaviors. However, some of The farmers’ green values influence the innovative green intention and green technology adoption.

Previously, no research has looked into the role of farmers’ green values in shaping their innovative green intentions. Therefore, this research fills the gap by exploring the role of farmers’ green values in their innovative green intentions. Moreover, there are some unanswered questions in this regard. There has been a conception in past that some farmers were hesitant to accept green, cutting-edge technology. This research dimension needed an exploration of the scope of adoption of green technology at large. Therefore, current research fills this gap in the literature and tries to answer the role of farmers’ green value on green technology adoption. The current work offers a novel explanatory strategy to address these problems, which are crucial for climate change adaptation and mitigation.

One highlighted moderator in this regard can regulate technology adoption behaviors in farmers. Health consciousness based on the health belief model (Ataei et al., 2021) provides vital support to the current research. Using chemicals and pesticides in conventional agriculture leads to disturbed health of farmers and end users. Once the farmers are aware of the health issues associated with chemical pesticides, health consciousness may arise (Ataei et al., 2021). Health-conscious farmers will get more support for developing innovative intentions leading to the adoption of green technologies (Shang et al., 2018; Japutra et al., 2021). Therefore, based on this assumption, health consciousness is utilized as a moderator between farmers’ green values, innovative green intentions, and green technology adoption. This research is a comprehensive approach to exploring technology adoption behaviors. This research helps in addressing the following questions.

RQ1: What role can farmers’ green value play in developing green innovative intentions and technology adoption?

RQ2: How can health consciousness regulate the function of farmers’ green values towards developing innovative intentions and technology adoption?

## Review of literature

### Theoretical underpinning

This study gets support from some of the theories and intends to fill in the gaps of literature by addressing the shortcomings. These theoretical models include Value belief norm theory (VBNT), Technology Acceptance Model (TAM), Theory of planned behaviors (TPB), Innovation Diffusion Theory (IDT), Unified Theory of Acceptance and Use of Technology (UTAUT), and Health belief model (HBM). VBNT draws on a wide range of value orientations. This theory has often been used in research on values and behavior. According to Rogers and Shoemaker (1971) and Davis (1989), innovativeness is the extent to which a person adopts a concept relatively early. Three fundamental theories are dominant in the research currently available for analyzing why innovations are accepted. There is a model for how people learn to accept and utilize new technologies called the TAM (Davis, 1989). When prospective users are shown a new and innovative technology, the model suggests that various variables influence their choice on whether or not to embrace it. These elements include the perceived utility and simplicity of use of the technology.

TPB (Ajzen, 1991) in the continuity of TAM, relates an individual’s beliefs and behaviors. Current research shows that farmers’ behaviors towards technology adoption can be tailored through TPB under TAM. TPB is often used as a theoretical model in research on the adoption behavior in general and innovations in specific. Attitude toward the activity, social impact on the behavior, and perceived behavioral control in executing the behavior are the three antecedents of behavioral intention in the TPB. The perceived behavioral control is the most important of these three (Ajzen, 1991). The IDT and the TAM are two theories of innovation adoption that may be added to the TPB. The IDT was first introduced by Rogers (1962). This theory holds the justification for the patterns of spread of products, new ideas and processes among the population. It elaborates the speed and pattern of the spread of these ideas, processes, and products in the target population. The TAM claims two elements influence how people feel about and adopt new technology (Davis, 1989). Many studies integrate TAM and TPB in agriculture sector for the development of farmers’ intentions toward adoption of green technologies (see, e.g., Wauters et al., 2010; Pierpaoli et al., 2013; Menozzi et al., 2015; Caffaro et al., 2020). This study also utilizes the basis of these theories to support the green innovative intentions and green technology adoption behaviors of farmers.

Attempting to explain user intents to utilize information technologies and subsequent use behavior, the UTAUT (Venkatesh et al., 2003) was developed. According to the theory, four primary factors impact a person’s choice to embrace a new practice: the expectation of performance, the expectation of effort, social influence, and enabling situations. The three different models were used to determine the factors that may lead to people’s adoption of specific technologies, such as computers and information networks. Furthermore, rather than farmers, the majority of the models emphasize consumer approval. Farmers confront particular obstacles to adoption yet are crucial for reducing climate change, adjusting to it, and ensuring the world’s food supply (Bukchin and Kerret, 2018).

Farmers’ intentions may be evaluated using behavioral models from the health sector which include but are not limited to TPB and HBM. The current research used the HBM in relation to TPB to examine farmers’ intentions towards their health consciousness as a planned behavior. The purpose of the current investigation is to determine the extent to which the dimensions of HBM (Health consciousness and the extended TPB (attitude, subjective norms, perceived behavioral control, self-identity, and moral norms) influence farmers’ intentions toward the use of environmentally friendly innovations (Ataei et al., 2021). Strategists may utilize the findings to create or modify plans to encourage farmers to use green insecticides. Farmers’ intentions and actions have been effectively explained and predicted by TPB in a variety of contexts. These contexts included the adoption of fish production, animal-friendly practices, agricultural output diversification, better natural grasslands, and chemical use (Borges et al., 2019; da Silva et al., 2020).

The HBM was presented firstly by Hochbaum et al. (1952) and is a well-established conceptual model used in the public health field, and it explains why people do not engage in preventive health initiatives (Willis, 2018). HBM is being utilized more and more in various disciplines, particularly agriculture and rural development. These include the consumption of organic food, water demand management, pesticide safety behavior, sustainable practices in gastrointestinal nematode control, and sustainable water management (Yazdanpanah et al., 2015; Moradhaseli et al., 2019; Moghadam et al., 2020). The current research utilizes all these theories to support the research model. These theories support the impact of farmers’ green values on green innovative intentions and green technology adoption behaviors. Moreover, health consciousness which is moderating these relationships gets strong support from HBM.

## Hypothesis development

### The role of farmers’ green values

Numerous instructional, outreach, incentive, and extension initiatives and regulations are designed to persuade farmers to adopt environmental techniques. The following are examples of focal practices: riparian buffers, forest set-asides, no-till or organic farming that is wildlife-friendly (Chapman et al., 2019). An alternate strategy looked at whether farmers’ pro-environmental values or cultures may explain their motives. TPB, which emphasizes the importance of beliefs in influencing behavior, has often been used in research on the significance of farmer attitudes (Chapman et al., 2019). Another line of research has looked at the function of values in environmental behavior since attitudes are often considered to be preceded by values. Value-Belief-Norm theory (Stern et al., 1999), which draws on a wide range of value orientations, has often been used in research on values and behavior.

Similar approaches have often been used in studies of farmers’ values, weighing nature-oriented values against production-oriented ones (Swagemakers et al., 2017). Recent research initiatives called the cultural revolution in agriculture concentrate on how social and cultural influences influence motives and behavior. Modern integrative work has proven the relevance of program fit with farmers’ needs and values. It has worked to connect attitudes, values, and culture with other elements (such as socioeconomic, operational, and financial restrictions). This study links behaviors, values, and culture with the other aspects (Sorice and Donlan, 2015).

The green values, which are associated with farmers’ beliefs and attitudes, can help develop innovative green intentions and green technology adoption behaviors. Most agricultural technological innovation studies emphasize the economic, social, and spatial factors that hinder farmers from adopting new practices and technologies (Feder and Umali, 1993) and examine adoption from an economic point of view, implying economic objectivity or efficient choice making (Chatterji, 2016). However, not all farmers accept innovations even when these hurdles are eliminated. Thus economic, social, and geographic constraints fall short of offering a whole explanation for farmers’ technology adoption. Numerous case studies provide anecdotal proof that economic forces fail to fully explain agricultural innovations’ acceptance or rejection (Reganold and Wachter, 2016).

For instance, data from Kenya reveals that poor adoption rates are often caused more by illiteracy and a lack of trust than by expenses. As a result, some farmers lack confidence that the innovative technology will work, even after learning the potential advantages (Eidt et al., 2012). Farmers’ values and adherence to current agricultural methods are other elements that affect the adoption rate (Alomia-Hinojosa et al., 2018). For instance, the extent to which farmers’ values are tied to their chosen way of life is related to how much they want to keep living that way, according to a study of producers of energy crops (Warren et al., 2016). Like how risk aversion and the degree of ambiguity around technology adoption may influence choices, read an overview of theoretical and empirical studies by Marra et al. (2003). The literature suggests that the following hypotheses should be tested to evaluate the role of farmers’ green values in technology adoption.

*H1*: Farmer green values have a positive association with innovative green intention.

*H2*: Farmer green values positively affect green technology adoption behavior.

### Role of green innovative intention

TPB examines a person’s behavioral intention to determine what that person will do; in other words, behavioral intention predicts what that person will do (Hwang et al., 2019). The behavioral belief-based framework, socially constructed belief-based structure, and control belief-based structure have all been used to build the TPB’s perspective. According to the TPB, people’s intentions are impacted by their perceptions of the existence or absence of elements that aid or obstruct the execution of an activity. These ideas might be supported by prior encounters with the activity or seeing others exhibiting the behavior (Aliabadi et al., 2020). This theory has three components: attitude, subjective standards, and a person’s sense of behavioral control. Beliefs about conduct, standards, and management services as the foundation for attitude, normative beliefs, and perceived behavioral control (Aliabadi et al., 2020).

An individual’s overall evaluation of behavior is referred to as their attitude toward that conduct. According to Ataei et al. (2022), subjective norms are a person’s assessment of whether other people believe she/he should engage in the conduct. The pressure one feels from the significant individuals in their life to engage in or refrain from specific conduct is referred to as subjective norms (Ataei et al., 2022). The third determinant of behavioral intention is perceived behavioral control. It displays a person’s opinion of how easy or difficult an activity is to carry out. According to this theory, a person chooses to engage in a behavior when they judge it to be positive (attitude), judge the behavior to be easy or difficult (perceived behavioral control), or judge the behavior to be under their volitional control (perceived behavioral control), and judge the behavior to be supported by significant others (subjective norms; Ataei et al., 2022).

Despite variances in the activity that was being studied, the techniques for collecting data, and the methodologies used to evaluate the data, studies have demonstrated that attitude, subjective norms, and perceived behavioral control are positively connected with farmers’ intentions (Bagheri et al., 2019; Rezaei et al., 2019; Aliabadi et al., 2020; Ataei et al., 2022). Adopting organic farming and cultivating genetically altered crops also necessitates the acquisition of new skills, which can only be done by people who can and are willing to learn these new techniques. Additionally, conceptual modeling and statistical findings imply that the refusal to adopt practical innovations may be related to substantial external effects (Rezaei et al., 2019) and the adopter’s social environment (including peer imitation behaviors). Last but not least, sure farmers appear to be prepared to give up income to embrace conservation methods (Naspetti et al., 2017).

The results indicated that ideology also plays a role in the development process of decision-making. Numerous research recommends adjusting policies to account for individual behavioral characteristics (see, e.g., Turaga et al., 2010; Triste et al., 2018). However, there is little empirical research on adopters (Turaga et al., 2010), and little is known about how farmers use technology. Given the dearth of novel research approaches and a comprehensive, robust and multidisciplinary viewpoint, several studies even contend that current research on adopting agricultural technology is useless (Sun et al., 2015; Triste et al., 2018). Furthermore, according to Marra et al. (2003), personal behavioral indications have often been misinterpreted or dealt with insufficiently in earlier studies. Based on the literature supporting for behavioral intentions of farmers, the following hypotheses were tested.

*H3*. Green innovative intention positively correlates with green technology adoption behavior.

*H4*. Green Innovative intention positively mediates the relationship between green farmer values and green technology adoption behavior.

### The moderating role of health consciousness

According to HBM theory, people are likelier to choose healthy activities when they desire to be healthy and think such habits will benefit and promote their health (Chen and Lin, 2018). HBM’s many training areas allow it to influence not just attitude modifications but also the continuation or cessation of a behavior (Xue et al., 2020). Health awareness and behavioral evaluation are the two aspects of health behavior that HBM focuses on. The elements of this model have also been expanded to include signals to action and self-efficacy in addition to these two aspects. The willingness of a person to be concerned about health concerns is known as self-efficacy (Green et al., 2020). The concept of self-efficacy was first introduced by Bandura (1977). In this context of being health conscious, Green et al. (2020) considers health consciousness a part of self-efficacy of individuals.

Self-efficacy relates to a person’s impression of how easy or difficult it is to carry out intentional conduct or the degree to which a person has conscious control over behavior (Williams and Rhodes, 2016). In addition to these HBM dimensions, studies have shown that farmers’ intentions to embrace innovative technology may also be influenced by socioeconomic and personal qualities (Meijer et al., 2015). Lee and Yun (2015) found that attitudes regarding organic foods impacted direct purchase intentions. Studies show an inverse association between farmers’ protective behavior in applying pesticides and perceived obstacles (Akter et al., 2018).

Much research has been conducted in organizational management, which states that health consciousness is a regulating factor of many processes. In the current scenario, it is established that health consciousness may regulate the functioning of farmers’ green values, green innovative intentions, and green technology adoption behaviors of farmers. Therefore, based on the significance of the concept of health consciousness, it is assumed that it may have a moderating effect. HBM also supports the notion that if farmers are conscious about their health, they will undoubtedly develop innovative green intentions, leading to green technology adoption behaviors. The authors developed the following hypotheses and tested their significance in this regard.

*H5*. Health consciousness moderates the relationship between green farmer values and innovative green intention.

*H6*. Health consciousness moderates the relationship between green farmer values and green technology adoption behavior.

**Figure 1:**
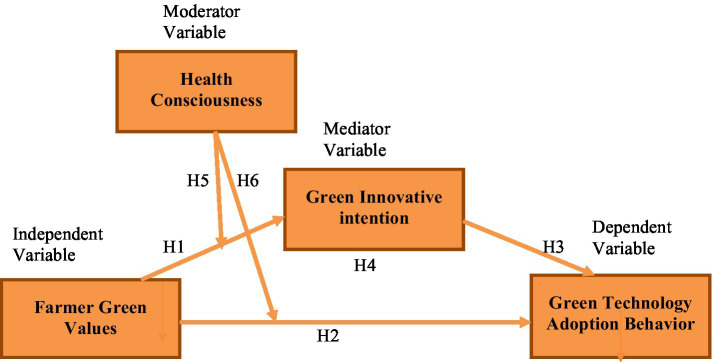
Conceptual framework.

The present study’s conceptual framework is given in Figure 1.

## Research methods

### Study design

The present study targeted farmers of grain production in China for data collection to evaluate their green behavior. The author collected data from farmers of grain production by applying a convenient sampling method. The author visited seed shops, met with their owners, and had a detailed meeting with them regarding the objective of data collection. The author explained to them the importance of the present study’s practical implication as this will be beneficial regarding environmental aspects. The author assured the owners of seed shops that the data would be gathered only for educational purposes instead of any marketing campaign. Finally, the owners of the seed shop permitted the author to collect data from their customers, such as farmers who came to buy grain seeds.

A cover letter was also developed along with questionnaires to explain the objective of the present study to the farmers. This cover letter assured the farmers of their data confidentiality and usage as it would be used only for academic purposes. The farmers had concerns that data could be used for author personal economic benefits or maybe for the complaint to authorities for not adopting or having green behavior. So this letter helped to reduce the farmers’ negative thoughts. Moreover, the cover letter also confident the farmers that no answers are wrong or right, so they choose the right and true answer. This way, the author tried to get as natural as possible responses from the farmers regardless of any social or official pressure.

The author developed questionnaires in English and also translated them into Chinese. As English is not very common among Chinese, the author took this initiative and developed dual-language questionnaires for the farmers’ easiness. For translation, the author got help from an expert in the Chinese language. Senior researchers also approved the translated questionnaires. Following the senior researchers’ guidance, the author also accumulated data from students to verify their Chinese language proficiency. This way, all errors were corrected, and senior researchers approved the final questionnaires for data collection.

The author took 3 months to collect data from farmers. The author sat at the shop from morning to evening for data collection from farmers. The author just requested the farmers to fill out the questionnaires and did not influence them while filling out questionnaires. The author targeted 400 farmers for data collection and received 322 responses. After scrutinizing the proper filling, the author found 303 responses appropriate for further processes such as data analyses. Hence, this study’s empirical examination is based on a 303 sample size.

### Measures

This study used five points Likert scale to measure the participants’ responses. This scale consists of five numbers where 1 means “strongly disagree,” 2 means “disagree,” 3 means “neutral,” 4 means “agree,” and 5 means “strongly agree.” This study considered previously validated items to assess the variables. Items can be seen in Appendix.

#### Farmers’ green values

The farmers’ green values construct was measured with 5 items scale adapted from Chou (2014) and Al-Ghazali and Afsar (2020). The sample item included, “I feel a personal obligation to do whatever I can to prevent environmental degradation.” The Cronbach alpha value is 0.884.

#### Green innovative intention

The green innovative intention was measured with 3 items scale of green behavior intention adapted from Norton et al. (2017) and Al-Ghazali and Afsar (2020). This scale was modified according to the innovative context. The sample item included, “I intend to show innovative environmentally friendly behavior at work.” The Cronbach alpha value is 0.829.

#### Green technological adoption behavior

The green technological adoption behavior was measured with 4 items scale adapted from Aboelmaged and Hashem (2019). The sample item included, “I adopt innovative technologies to minimize the environmental risks.” The Cronbach alpha value is 0.905.

#### Health consciousness

Health consciousness was measured with nine items scale adopted from Gould (1990). A sample item included, “I’m very self-conscious about my health.” The Cronbach alpha value is 0.896.

## Results

### Assessment of measurement and structural model

Structural equation modeling (SEM) is considered an appropriate statistical model for data analyses. Covariance-based (CB-SEM) and variance-based partial least squares structural equation modeling (PLS-SEM) are two different types of SEM (Hair et al., 2019a). The key difference in both methods is that CB-SEM is considered for theory acceptance and rejection, while PLS-SEM is considered for advancing and developing the theories (Hair et al., 2017; Bashir et al., 2021). The present study applied the PLS-SEM technique for data analysis. The key rationale behind this selection is the effectiveness of PLS-SEM for both confirmatory and exploratory studies (Hair et al., 2011). PLS-SEM is a useful approach for complex and multi-orders-based models and needs no specific data normality conditions. PLS-SEM is also suitable for evaluating small data sets (Hair et al., 2017). Therefore, this study considers the PLS-SEM method for empirical data analyses using Smart PLS 3.3.3 software. The outcomes of PLS-SEM-based analysis are estimated in two stages, including model measurement and structural model evaluation. The measurement model stage assesses the reliability and validity of the constructs, whereas the structural model examines the relationship between the proposed hypotheses. The acceptance or rejection of a hypothesis is evaluated through the “*t*” statistic and “*p*” values.

The model measurement outcomes are comprised of two parts: model reliability and validity. The present study considered the values of “Cronbach’s alpha, roh-A, composite reliability, and average variance extract (AVE)” to authenticate the model’s reliability (Hair et al., 2017), and all values are shown in Table 1. The values of Cronbach’s alpha are accepted if they are larger than 0.7 (Hair et al., 2017). Similarly, the value of composite reliability should also be greater than 0.7. All Cronbach’s alpha and composite reliability values are according to acceptable criteria, which is a positive indicator of the model’s reliability. The values of ROH-A reliability (0.887, 0.831, 0.908, 0.904) are also according to the acceptable criteria (Hair et al., 2017). The average variance extracts (AVE) values greater than 0.5 are considered good for the convergent validity of the model. The Table 1 shows that the AVE values of all constructs (0.684, 0.746, 0.778, and 0.657) are according to acceptable criteria.

**Table 1:** Reliability and convergent validity of the study constructs.

Construct	Item	Outer loadings	VIF	Alpha	roh-A	Composite reliability	AVE
FGV	FGV1	0.833	2.203	0.884	0.887	0.915	0.684
	FGV2	0.788	1.886				
	FGV3	0.837	2.305				
	FGV4	0.882	2.943				
	FGV5	0.791	2.052				
GII	GII1	0.831	1.767	0.829	0.831	0.898	0.746
	GII2	0.909	2.486				
	GII3	0.849	1.923				
GTAB	GTAB1	0.859	2.499	0.905	0.908	0.933	0.778
	GTAB2	0.900	3.100				
	GTAB3	0.907	3.179				
	GTAB4	0.861	2.443				
HC	HC1	0.791	2.211	0.896	0.904	0.920	0.657
	HC2	0.778	2.056				
	HC3	0.791	2.266				
	HC4	0.823	2.242				
	HC5	0.837	2.452				
	HC6	0.842	2.514				

FGI, Farmer Green Values; GII, Green Innovative Intention; GTAB, Green Technology Adoption Behavior; HC, Health Consciousness.

**Figure 2:**
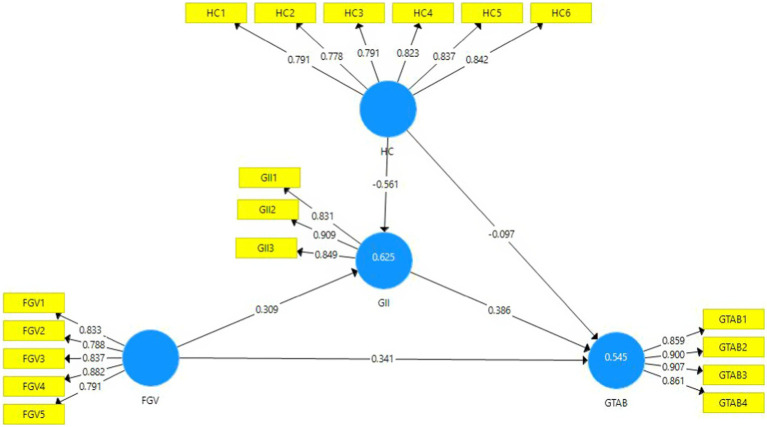
Path estimates and outer loadings.

Table 1 explains that the current study’s framework is based on 18 items of the four variables. All items’ outer loading values of models’ constructs are shown in Table 1. The outer loading values of items are considered reliable if they are greater than 0.7 (Hair et al., 2017). Table 1 depicts that the outer loading values of all items are according to the required criteria (Figure 2). The VIF values are evaluated to confirm the collinearity issues in the model. The model is considered free from collinearity issues if the VIF values are below 0.5 (Hair et al., 2011). According to the results in Table 1, all VIF values are less than 0.5, such as the variable “green technology adoption behavior” item GTAB-3 has the highest VIF value (3.179). Hence, it is verified that the model of the present study is free from collinearity issues.

The *R*^2^ values are evaluated to define the model’s strength, such as the values of latent variables greater than or near 0.5 indicating moderate strength of the model, and the values near 0.25 showing weak model strength (Hair Jr et al., 2014). The *R*^2^ values of the endogenous variables of the present study model (green innovation intentions and green technology adoption behavior) are 0.625 and 0.545, respectively, which shows moderate model strength (Hair et al., 2017). The cross-validated redundancy (*Q*^2^) values of the model are considered significant if they are greater than zero (Hair Jr et al., 2014). The *Q*^2^ values of all latent variables of the current study are greater than zero, which is another positive sign of model significance.

Fornell–Larcker criterion and heterotrait–monotrait (HTMT) ratios are well-known approaches that examine the discriminant validity of the model’s constructs (Hair et al., 2017). The present study used these two approaches for assessing constructs’ validity. The Fornell–Larcker criterion is measured by taking the square roots of AVE values of model variables (Hair Jr et al., 2014). The Fornell–Larcker criterion values of constructs are presented in Table 2. The values under the Fornell–Larcker criterion are accepted if the upper side first value of each column is greater than their below values. Table 2 shows that all values of the Fornell–Larcker criterion are according to the accepted criteria. Hence, it is verified that discriminant validity established on the Fornell–Larcker criterion has been achieved in this study. In addition, according to the specified criteria, the HTMT values of all variables should be less than 0.85; however, values greater than 0.90 are also acceptable (Hair et al., 2019b). According to the outcomes (Table 3), the HTMT values of constructs are less than 0.85, which confirmed that discriminant validity in the current study’s model has been established.

**Table 2:** Discriminant validity (Fornell-Larker-1981 Criteria).

Construct	FGV	GII	GTAB	HC
FGV	**0.827**			
GII	0.657	**0.864**		
GTAB	0.655	0.683	**0.882**	
HC	−0.620	−0.753	−0.599	**0.811**

FGI, Farmer Green Values; GII, Green Innovative Intention; GTAB, Green Technology Adoption Behavior; HC, Health Consciousness. The bold values are the results for corresponding statistics for whole variable not the items.

**Table 3:** Discriminant validity (HTMT).

Construct	FGV	GII	GTAB	HC
FGV	–	–	–	–
GII	0.767	–	–	–
GTAB	0.730	0.788	–	–
HC	0.689	0.860	0.658	–

FGI, Farmer Green Values; GII, Green Innovative Intention; GTAB, Green Technology Adoption Behavior; HC, Health Consciousness.

### Hypotheses testing

The empirical examination of the present study is conducted by using 5,000 samples of the bootstrapping method (Hair Jr et al., 2014; Hair et al., 2017). The results of the direct, indirect, and total paths are depicted in Table 4. The current study considered the “*t*” values and “*p*” values of statistics for the acceptance and rejection of hypotheses. Table 5 shows the results of the hypotheses proposed by the present study. The outcomes of the first hypothesis (*t* = 5.432, *p* = 0.000) confirmed that farmers’ green values have a positive association with green innovative intentions. The beta value of hypothesis 1 confirmed that one unit change in the independent variable (farmers’ green values) would result in 0.268 changes in the dependent variable (green innovative intentions). Hence hypothesis 1 of the present study is accepted. The findings of the second hypothesis (*t* = 5.685, *p* = 0.000) confirmed that farmers’ green values have a positive association with green technology adoption behavior. The beta value of H2depicted that one unit change in the independent variable (farmers’ green values) would result in 0.337 changes in the dependent variable (green technology adoption behavior). Hence the second hypothesis of the present study is also accepted. The findings of the third hypothesis (*t* = 5.493, *p* = 0.000) confirmed that green innovative intention has a positive association with green technology adoption behavior. Moreover, the beta value indicated that one unit change in the independent variable (green innovative intention) would result in 0.379changes in the dependent variable (green technology adoption behavior). Hence the H3 of the present study is also accepted.

**Table 4:** Direct, indirect, and total path estimates.

	Beta	SD	*t*	*p*
*Direct path*				
FGV→GII	0.268	0.049	5.432	**0.000**
FGV→GTAB	0.337	0.059	5.685	**0.000**
FGV*HC→GII	0.069	0.019	3.685	**0.000**
FGV*HC→GTAB	0.011	0.019	0.591	**0.555**
GII→GTAB	0.379	0.069	5.493	**0.000**
HC→GII	−0.453	0.056	8.035	**0.000**
HC→GTAB	−0.084	0.064	1.320	**0.187**
*Indirect path*				
FGV→GII→GTAB	0.102	0.026	3.879	**0.000**
FGV*HC→GII→GTAB	0.026	0.009	3.054	**0.002**
HC→GII→GTAB	−0.172	0.037	4.678	**0.000**
*Total path*				
FGV→GII	0.268	0.049	5.432	**0.000**
FGV→GTAB	0.439	0.063	6.970	**0.000**
FGV*HC→GII	0.069	0.019	3.685	**0.000**
FGV*HC→GTAB	0.026	0.009	3.054	**0.002**
FGV*HC→GTAB	0.011	0.019	0.591	**0.555**
GII→GTAB	0.379	0.069	5.493	**0.000**
HC→GII	−0.453	0.056	8.035	**0.000**
HC→GTAB	−0.256	0.062	4.131	**0.000**

FGI, Farmer Green Values; GII, Green Innovative Intention; GTAB, Green Technology Adoption Behavior; HC, Health Consciousness. The bold values are the results for corresponding statistics for whole variable not the items.

**Table 5:** Hypotheses testing.

		Coefficient (Beta)	S.D	*t*	*p*	Status
*Hypotheses*						
H1	FGV→GII	0.268	0.049	5.432	0.000	Supported
H2	FGV→GTAB	0.337	0.059	5.685	0.000	Supported
H3	GII→GTAB	0.379	0.069	5.493	0.000	Supported
*Mediation hypotheses*						
H4	FGV→GII→GTAB	0.102	0.026	3.879	0.000	Supported
*Moderation hypotheses*						
H5	FGV*HC→GII	0.069	0.019	3.685	0.000	Supported
H6	FGV*HC→GTAB	0.011	0.019	0.591	0.555	Not supported

FGI, Farmer Green Values; GII, Green Innovative Intention; GTAB, Green Technology Adoption Behavior; HC, Health Consciousness.

The present study also assumes the mediating role of green innovative intention in the relationship between farmer green values and green technology adoption behavior. For the empirical investigation of mediating role, the present study assumes H4. According to findings (*t* = 3.879, *p* = 0.000), green innovative intention positively mediates in the relationship between farmer green values and green technology adoption behavior, and the path value of H4 is (0.102). Hence, it is confirmed that the fourth hypothesis of the present study is accepted.

The present study also evaluated the moderating role of health consciousness in the relationship between farmer green values and green innovative intention, and between farmer green values and green technology adoption behavior, respectively. For empirical investigation present study proposes H5 and H6. The results of H5 (*t* = 3.685, *p* = 0.000) confirmed that health consciousness moderates the relationship between farmer green values and green innovative intention. Hence, the fifth hypothesis of the present study is accepted. The outcomes of H6 (*t* = 0.591, *p* = 0.555) revealed that health consciousness does not moderate the relationship between farmer green values and green technology adoption behavior. Therefore, the sixth hypothesis of the present study is rejected.

## Discussion

The rapid development of China’s economy leads to severe ecological problems, including climate changes, natural resource depletion, and environmental pollution (Duan et al., 2021). The state successively introduced a chain of ecological restoration projects to deal with these ecological complications, and the Grain for Green Project (GGP) is one of them. The key purpose of this Program is to decrease soil erosion and to develop ecological conditions (Zhou et al., 2012). The agricultural sector must vigorously promote green innovation and green production among farmers to achieve the targets of ecological restoration projects. Additionally, it is noticed that farmers’ green values could play a bridging role because the farmers with green values exhibit a green attitude and behavior toward farming (Al-Ghazali and Afsar, 2021).

Lioutas and Charatsari (2018) argue that various motivational pathways might encourage farmers to adopt green innovations, including “environmental concern, convenience, economic incentives and the internal need to pursue change.” However, green technology adoption could be an important means of dealing with environmental issues. The adoption of green technology could enrich economic and environmental performance. The present study aims to find the association of farmers green values and green technology adoption behavior. For empirical investigation, the current study hypothesized that farmer’s green values have a positive association with green innovative intention and green technology adoption behavior, respectively. Further, this study assumes that green innovative intention positively impacts green technology adoption behavior. The present study also aims to determine the mediating role of green innovative intention in the relationship between farmer green values and green technology adoption behavior. This study also attempts to check the moderating role of health consciousness in the relationship between farmer green values and green innovative intention, and the relationship between farmer green values and green technology adoption behavior, respectively.

The present study discovered that farmers’ green values positively correlate with green innovative intentions, which means the first hypothesis is accepted. These findings have consistency with prior studies (Adnan et al., 2018; Cheema et al., 2020; Al-Ghazali and Afsar, 2021). These studies argue that individual values are crucial in creating and generating innovative ideas. Further, they acknowledged that the green values shape the attitude and behavior of individuals toward innovative intentions. Farmers’ green values motivate them to build intentions for green innovation ideas for farming. The present study further revealed that farmers’ green values positively affect green technology adoption behavior. The prior studies also argue about the importance of farmers’ green values for green technology adoption behavior (Gao et al., 2020; Xia et al., 2021). Additionally, Marvuglia et al. (2022) noticed that effective environmental management arises when individual and their workplace green values have consistency. Green values are likely to influence people’s in-role and extra-role behavior.

The current study’s findings further acknowledged that green innovative intention positively impacts green technology adoption behavior, which means that the third hypothesis is also accepted. These findings are consistent with previous studies (Bukchin and Kerret, 2018; Adnan et al., 2019; Mao et al., 2021). According to these studies, the green innovation intentions build the behavior of individuals to adopt green technology. Further, they argue that farmers’ green technology adoption behavior plays an important role in boosting the productivity of the agriculture sector. The present study also assessed the mediating role of green innovative intention in the relationship between farmers’ green values and green technology adoption behavior. The findings confirmed that green innovative intention positively mediates the relationship between farmer green values and green technology adoption behavior. Mao et al. (2021) point out that farmers surely play a significant role in the development of green agriculture as they are primary decision-and action-makers in agricultural production. However, farmer characteristics and values influence how farmers embrace green agriculture technologies.

The present study also assumes the moderating role of health consciousness in the relationship between farmers’ green values and green innovative intention and between farmer green values and green technology adoption behavior. The results confirmed that health consciousness moderates the relationship between farmers’ green values and green innovative intention. However, the results revealed that health consciousness does not moderate the relationship between farmers’ green values and green technology adoption behavior. The prior studies also point out that health-related concerns and economic incentives are important factors that might motivate farmers to adopt green innovation and green technology behaviors (Wang et al., 2019; Elahi et al., 2022).

### Theoretical and practical implications

The results indicated that current research has several implications for the agricultural community. Firstly, it confirms the processes involved in the theory of planned behaviors. The impact of farmers’ green values on their innovative intentions and technology adoption behaviors has a profound standing for future research. Once farmers have developed strong green values, they will develop innovative intentions. These innovative intentions are purposefully contributing to developing technological behaviors among farmers. Farmers’ behaviors are being shaped up towards adoption of technology. Therefore, the findings of this research have strong links with the theory of planned behaviors. Similarly, some previous researchers also confirmed the significance of the theory of planned behaviors in technology adoption (Borges et al., 2019; da Silva et al., 2020). It indicates that certain behaviors can be planned among individuals.

Furthermore, this research contends that farmers would adopt technology when they have solid green values. This would allow them to accept the innovative ideas and use of technology to achieve the targets of innovations. This also confirms that future researchers can get support from technology acceptance model in a way that farmers may develop their values around the usefulness of tools involved in technology adoption. This study is in line with technology acceptance model as it indicates that innovative intentions force the farmers to adopt green technologies in their farming patterns due to the usefulness of the technology. Lastly, this study has implications for the health belief models.

The role of health consciousness indicates that farmers’ belief in health supports the development of innovative intentions, which lead to the technology adoption behaviors. The HBM indicates that intentions lead to behavioral approaches of the individuals. Therefore, health consciousness shows a regulating role in shaping the innovative intentions toward behavioral development of green technology adoption. Moreover, this research implies that the farming community should be directed towards adopting technological innovations that would safeguard end users’ health and well-being. It would also contribute to the financial status of the farming community. This study implies that farm management should be brought into practice for the adoption of innovative technologies which would preserve the environment. This study also directs the environmentalists to develop innovative technologies which are farmer and environment-friendly as well as economical.

## Limitations and future research directions

The present study serves the literature in multiple ways, but still, there are some gaps, which may become opportunities for scholars to conduct their research in the future. First, the present study assumes farmers’ green values as an antecedent of green innovative intention and green technology adoption behavior; future studies may consider other possible antecedents such as environmental concerns, economic pressure, etc. Al-Ghazali and Afsar (2021) also point out that environmental concerns and economic pressure could pave the way for development of green intensions and values in farmers. Second, this study assessed the mediating role of green innovative intentions; future studies may consider some other mediating variables like commitment and engagement, etc. The green values positively developed in farmers when they have engagement and commitment for green innovation and technology adoption behaviors (Mao et al., 2021). Fourth, this study is conducted using a small sample size; in the future, researchers may extend the sample size to authenticate the present study’s model. Fifth, this study is conducted in China, and the results may not be generalizable to other contexts. Scholars in the future may conduct the same study in other developing or developed countries for a better understanding of the results. Finally, this study collected the data using a structured questionnaire method; in the future, scholars may use other data collection methods such as semi-structured questionnaires, interview methods, etc.

## Conclusion

Farmers’ green values could play a constructive role in building green innovative intention and green technology adoption behaviors. The adoption of green technology could enrich economic and environmental performance. The present study determines the association between farmers’ green values and green technology adoption behavior. For empirical investigation, the current study assumes that farmer’s green values positively correlate with green innovative intention and green technology adoption behavior, respectively. Additionally, this study proposes that green innovative intention positively correlates with green technology adoption behavior. The present study also attempts to determine the mediating role of green innovative intention and the moderating role of health consciousness. The current study confirmed that farmers’ green values positively impact green innovative intention and green technology adoption behavior, respectively. Further, it is also verified that green innovative intention positively correlates with green technology adoption behavior. The finding also authenticated that green innovative intention positively mediates the relationship between farmer green values and green technology adoption behavior. The moderating role of health consciousness in the relationship between farmer green values and green innovative intention is also confirmed by the results of this study. However, the findings revealed that health consciousness does not moderate the relationship between farmers’ green values and green technology adoption behavior. The findings of current investigation serve the literature by pointing out the importance of farmers green values to build their intentions for green innovation. Further the outcomes of current study acknowledged that farmers will adopt creative aims after developing strong green intentions and values and these creative goals are consciously fostering the emergence of technological behaviors among farmers. The findings further highlighted the importance of health consciousness and suggest that farmers’ commitment to their well-being encourages the creation of creative intents that result in technological adoption behaviors.

## Data availability statement

The original contributions presented in the study are included in the article/supplementary material, further inquiries can be directed to the corresponding author.

## Author contributions

RG conceived the idea. HZ designed the manuscript. CG wrote the paper. ZW revised the document. All authors read and agreed to the published version of the manuscript.

## Funding

This study was supported by the National Social Science Fund Project “Research on the Linkage and Integration Mechanism of the Household Registration System and the Agricultural Land Property Rights System under the Background of Urban-rural Integrated Development” (Project No: 19BJY120). It was also supported by Jiangxi Association for Science and Technology’s 2018 decision-making consultation project “Practical Exploration and Policy Suggestions on the Cultivation of New Professional Farmers in Jiangxi Province” (Gan Ke Xie Zi [2018] No. 115) and the Ministry of Education’s Humanities and Social Sciences Research Youth Fund Project “Research on the Adoption Behavior of Rice Farmers’ Specialized Unified Prevention and Control: Bias Recognition, Transformation Mechanism and Diffusion Effect” (Project No: 21YJC790127). Research on the adoption behavior and guiding policies of farmers’ specialized unified defense and governance in southern rice farming areas (Project No: JJ20211).

## Conflict of interest

The authors declare that the research was conducted in the absence of any commercial or financial relationships that could be construed as a potential conflict of interest.

## Publisher’s note

All claims expressed in this article are solely those of the authors and do not necessarily represent those of their affiliated organizations, or those of the publisher, the editors and the reviewers. Any product that may be evaluated in this article, or claim that may be made by its manufacturer, is not guaranteed or endorsed by the publisher.

## Appendix

**Measurement items**

### Green values

I feel a personal obligation to do whatever I can to prevent environmental degradation.
I feel obliged to save environment from degradation, regardless of what others do.
People like me should do whatever they can to protect environment from degradation.
I feel guilty when I contribute in environmental degradation.
I feel obliged to bear the environment and nature in mind in my daily behavior.

#### Green innovative intentions

I intend to show innovative environment friendly behavior at work.
I plan to act in an innovative environment friendly way.
I intend to show environment friendly behavior at work.

#### Green technological adoption behavior

I adopt innovative technologies to minimize the environmental risks.
I adopt cleaner technologies.
I try to reuse or recycles inputs, materials and wastes.
I can substitute toxic materials with eco-friendly ones.

#### Health consciousness

I reflect about my health a lot.
I’m very self-conscious about my health.
I’m generally attentive to my inner feeling about my health.
I’m constantly examining my health.
I’m alert to changes in my health.
I’m usually aware of my health.
I’m aware of the state of my health as I go through the day.
I notice how I feel physically as I go through the day.
I’m very involved with my health.
